# PfCAP-H is essential for assembly of condensin I complex and karyokinesis during asexual proliferation of *Plasmodium falciparum*

**DOI:** 10.1128/mbio.02850-23

**Published:** 2024-04-02

**Authors:** Pratima Gurung, James P. McGee, Jeffrey D. Dvorin

**Affiliations:** 1 Division of Infectious Diseases, Boston Children's Hospital, Boston, Massachusetts, USA; 2 Department of Pediatrics, Harvard Medical School, Boston, Massachusetts, USA; The George Washington University Milken Institute of Public Health, Washington DC, USA

**Keywords:** *Plasmodium*, mitosis, chromosome, condensin I, karyokinesis

## Abstract

Condensin I is a pentameric complex that regulates the mitotic chromosome assembly in eukaryotes. The kleisin subunit CAP-H of the condensin I complex acts as a linchpin to maintain the structural integrity and loading of this complex on mitotic chromosomes. This complex is present in all eukaryotes and has recently been identified in *Plasmodium* spp. However, how this complex is assembled and whether the kleisin subunit is critical for this complex in these parasites are yet to be explored. To examine the role of PfCAP-H during cell division within erythrocytes, we generated an inducible PfCAP-H knockout parasite. We find that PfCAP-H is dynamically expressed during mitosis with the peak expression at the metaphase plate. PfCAP-H interacts with PfCAP-G and is a non-SMC member of the condensin I complex. Notably, the absence of PfCAP-H does not alter the expression of PfCAP-G but affects its localization at the mitotic chromosomes. While mitotic spindle assembly is intact in PfCAP-H-deficient parasites, duplicated centrosomes remain clustered over the mass of unsegmented nuclei with failed karyokinesis. This failure leads to the formation of an abnormal nuclear mass, while cytokinesis occurs normally. Altogether, our data suggest that PfCAP-H plays a crucial role in maintaining the structural integrity of the condensin I complex on the mitotic chromosomes and is essential for the asexual development of malarial parasites.

**IMPORTANCE:**

Mitosis is a fundamental process for *Plasmodium* parasites, which plays a vital role in their survival within two distinct hosts—human and *Anopheles* mosquitoes. Despite its great significance, our comprehension of mitosis and its regulation remains limited. In eukaryotes, mitosis is regulated by one of the pivotal complexes known as condensin complexes. The condensin complexes are responsible for chromosome condensation, ensuring the faithful distribution of genetic material to daughter cells. While condensin complexes have recently been identified in *Plasmodium* spp., our understanding of how this complex is assembled and its precise functions during the blood stage development of *Plasmodium falciparum* remains largely unexplored. In this study, we investigate the role of a central protein, PfCAP-H, during the blood stage development of *P. falciparum*. Our findings reveal that PfCAP-H is essential and plays a pivotal role in upholding the structure of condensin I and facilitating karyokinesis.

## INTRODUCTION

*Plasmodium falciparum* is a protozoan parasite that is responsible for the most severe forms of human malaria. Malaria remains one of the most important global infectious diseases, claiming more than 619,000 lives worldwide annually (1). These parasites have a complex life cycle that alternates between two different hosts, *Anopheles* mosquitoes and humans. To survive and thrive in these hosts, they replicate through an atypical cell division. These parasites follow schizogony within hepatocytes and red blood cells (erythrocytes) in their human hosts. On the other hand, they undergo gametogenesis and sporogony in mosquitoes (2). Among all these stages, the extensive proliferation of parasites within human erythrocytes causes the signs and symptoms of clinical malaria. Parasite cell division in erythrocytes, known as schizogony, is an unconventional mode of cell division that includes growth and budding phases (3, 4). During the growth phase, the parasite undergoes several asynchronous rounds of DNA replication and mitosis (S–M phase) without cytokinesis (5, 6). Asexual *Plasmodium* parasites undergo closed mitosis, where decondensed chromosomes are segregated, followed by nuclear division with an intact nuclear envelope throughout the cycle (2). Later in the budding phase, the parasite undergoes a final-round semi-synchronous nuclear division along with cytokinesis to produce mature daughter cells, called merozoites. These merozoites invade new erythrocytes to begin the proliferation cycle again (7, 8). Despite its predominant role in asexual proliferation, the regulation of this atypical mitosis is still underexplored in *Plasmodium* parasites.

During mitosis, chromatin condensation and segregation are important events to ensure that genomic material is equally divided into the daughter nuclei (9, 10). This process is mediated by two distinct condensin complexes, condensin I and II. These complexes are pentameric and comprised of two parts—core subunits and regulatory subunits. The core subunit consists of structural maintenance of chromosomes (SMC) 2 and 4, common in condensin I and II complexes. In contrast, the regulatory subunits, collectively known as chromosome-associated proteins (CAP) or non-SMC members, differ between two complexes; condensin I contains CAP-H/CAP-G/CAP-D2 while condensin II has CAP-H2/CAP-G2/CAP-D3 (11, 12). While condensin I is highly conserved across eukaryotes, and its localization is dynamic throughout the cell division, condensin II remains in the nucleus and is absent in some organisms (e.g., yeast and insects) (12, 13).

In *Plasmodium* spp., both condensin I and II complexes were identified by homology prediction (14, 15). The two core subunits (SMC 2/4) and one non-SMC member PfCAP-G of the condensin I complex have been genetically interrogated in *Plasmodium* spp. (14, 16). In *Plasmodium berghei*, SMC2/4 displayed a dynamic localization in both asexual parasites and gametocytes. PbSMC2/4 knockout in asexual parasites was unsuccessful; therefore, a functional evaluation was not possible during that stage. Depletion of PbSMC2/4 during gametocytogenesis impaired male gametogenesis and zygote differentiation and thus blocked parasite transmission in *P. berghei* (14).

On the other hand, the knockdown of PfCAP-G [or Merozoite Organizing Protein (MOP)], a non-SMC member of the condensin I complex, showed a fitness defect in the asexual development of *P. falciparum*. The PfCAP-G-deficient parasites showed flawed segmentation with a large residual agglomerate of partially divided cells (16). Given that the PfCAP-G knockdown phenotype was incomplete, likely due to insufficient protein knockdown, the phenotype of complete loss of condensin I remains insufficiently evaluated. Furthermore, there remains a lack of experimental evidence on how these complexes are assembled on chromosomes and their function as regulators of mitosis during the asexual blood stage development in *P. falciparum*.

In eukaryotes, the kleisin subunit CAP-H acts as a linchpin in the assembly of the condensin I complex (17). The CAP-H sequence consists of five motifs that bind to different components of the condensin I complex (18–20). The N- and C-terminal motifs of this protein interact with the core proteins (SMC 2 and SMC 4), while the central regions consist of motifs that bind to two other non-SMC proteins, CAP-G and CAP-D2, along with a region that interacts with chromosomal DNA to anchor condensin (18, 21–23). In addition, the loading of the condensin I complex during mitosis is regulated by the N-terminal tail of CAP-H in *Xenopus* egg extracts (24). Mutation or deletion of CAP-H results in a mitotic chromosome condensation and segregation defect in yeast and *Drosophila melanogaster* (25–27).

CAP-H has been bioinformatically predicted in *Plasmodium* spp. (14), and we have focused on interrogating its function during the asexual development of *Plasmodium falciparum* within human erythrocytes. We generated a parasite strain allowing inducible knockout of PfCAP-H. We show that PfCAP-H is a member of the condensin I complex and is essential for asexual replication of the parasite. PfCAP-H is dynamically localized during mitosis and can be used as a marker for the metaphase plate. Depletion of PfCAP-H causes abnormal karyokinesis, while cytokinesis occurs normally. This study provides new insights into the function of the condensin I complex during asexual replication of *P. falciparum*.

## RESULTS

### PfCAP-H has conserved N- and C-terminal regions and is expressed in proliferative blood stages

CAP-H is a central component of the condensin I complex in all eukaryotes (17). Bioinformatic analysis predicted that CAP-H is also present in *Plasmodium falciparum* (14). PF3D7_1304000 (hereafter referred to as PfCAP-H) is 1,024 amino acids long with a putative condensin complex subunit 2 domain. To comprehensively evaluate the PfCAP-H sequence by *in silico* analysis, we compared the sequence of PfCAP-H with CAP-H homologs from a wide range of eukaryotes. PfCAP-H consists of conserved regions in its N (1–250 aa) and C (890–1,000 aa) termini, with a less conserved interior sequence (Fig. S1A). The complete amino acid sequence has 21%–34% similarity with the majority of the non-Apicomplexan homologs and, as expected, higher similarity to *Plasmodium* homologs (e.g., 64% to PbCAP-H) (Fig. S1B).

**Fig 1:**
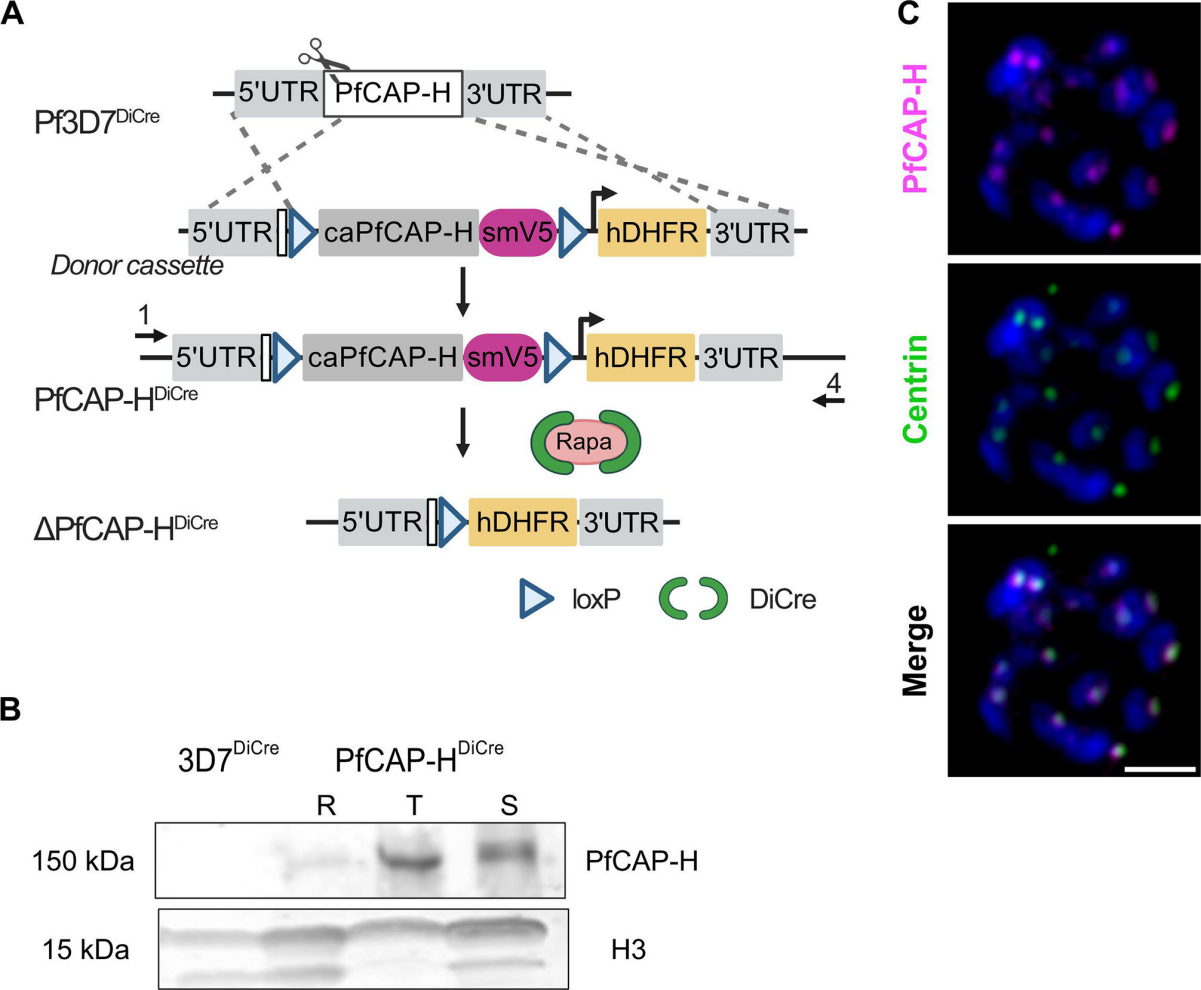
Expression and localization of PfCAP-H in PfCAP-H^DiCre^ parasites. (**A**) Schematic of PfCAP-H^DiCre^ parasites. In PfCAP-H^DiCre^ parasites, we replaced the endogenous locus of *PfCAP-H* in 3D7*pfs47*DiCre (referred to as 3D7^DiCre^) parasites with a loxP-flanked (triangle), codon-altered PfCAP-H with spaghetti monster (sm)-V5 tag (magenta-colored box) at the C-terminus. (**B**) Immunoblot showing the expression of PfCAP-H during asexual blood stages (R, rings; T, trophozoites; S, schizonts) probed with α-V5. The parental Pf3D7^DiCre^ parasites are used as a negative control, and α-Histone H3 was used as a protein loading control. (**C**) The localization of PfCAP-H was visualized by α-V5 (magenta) for smV5-tagged PfCAP-H, and α*-*Centrin (green) was used as a marker of centrosome by slide-based immunofluorescence assay (IFA). The IFA showed that PfCAP-H is localized near centrosome. The DNA was stained with Hoechst 33342 (blue). Scale bar = 2 µm.

To directly investigate the role of PfCAP-H in *Plasmodium falciparum*, we generated an inducible PfCAP-H knockout (iKO) strain in 3D7*pfs47*DiCre parasites (named PfCAP-H^DiCre^) (28) (Fig. 1A). In these parasites, the native PfCAP-H gene locus has been replaced with a *loxP*-flanked, codon-altered PfCAP-H with the spaghetti monster (sm)V5 epitope tag at the C-terminus. Integration of the donor cassette was verified by PCR amplification. The expected sizes of 2.3 and 0.98 kilobase-pairs (kb) were observed in the transgenic line, while no equivalent bands were amplified in the parental line (Fig. S2A).

We next sought to determine the expression timing and localization of PfCAP-H during the asexual blood stages. The immunoblot, probed with *α*-V5, demonstrated the expected 150 kDa of smV5-tagged PfCAP-H in both trophozoites and schizonts (Fig. 1B). The observed PfCAP-H expression agrees with the transcriptional profiling data (15), indicating a likely role for PfCAP-H during asexual development. Given that PfCAP-H homologs are present near centrosomes in other organisms (12), we performed an IFA on schizonts with an antibody that recognizes *Plasmodium* centrins as a marker for the centrosome in these parasites. PfCAP-H localizes near the centrosomes (Fig. 1C), similar to the reported localizations for PbSMC2/4 (14).

### PfCAP-H is highly expressed in mitotically active nuclei during schizogony

To interrogate the subcellular localization of PfCAP-H during the proliferative stages of asexual development, we performed IFAs throughout the schizont stage of the asexual development cycle, probing for PfCAP-H (*α*-V5) together with the nuclear (DNA) stain Hoechst 33342. The IFA revealed discrete perinuclear foci throughout schizogony (Fig. S3), with diminished protein detection when segmentation is complete. This expression resembles the pattern of proteins crucial for cell cycle progression (29).

**Fig 2:**
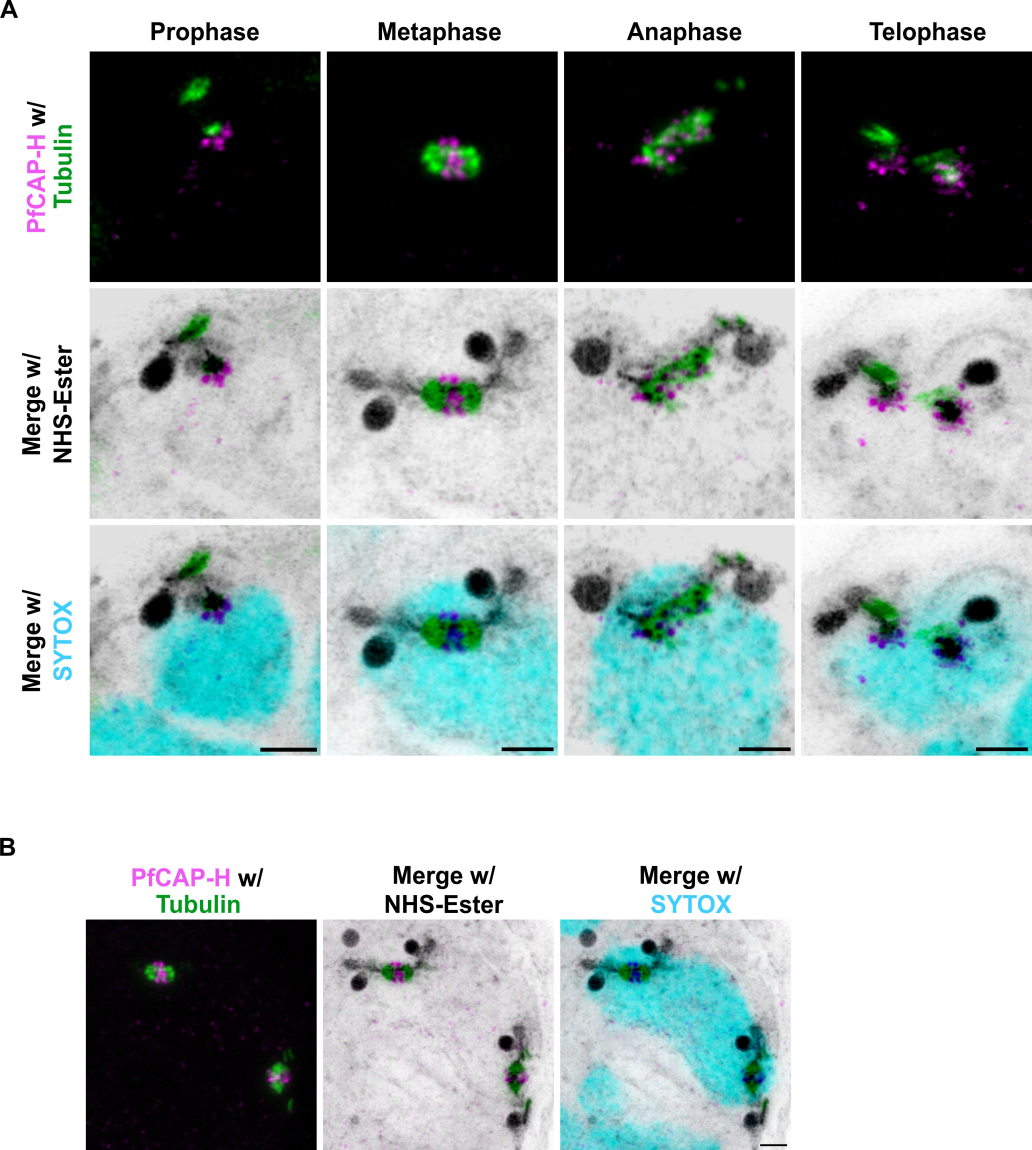
PfCAP-H is present in mitotically active nuclei. (**A**) PfCAP-H^DiCre^ parasites were prepared for U-ExM and stained with α-V5 for PfCAP-H (in magenta), α-tubulin for mitotic spindles (in green), NHS-Ester for protein stain (in grayscale), and SYTOX for nuclear stain (in cyan) and captured using Airyscan microscopy. With the cues from the localization of mitotic spindles, different stages of mitosis (prophase, metaphase, anaphase, and telophase) are marked compared with position of mitotic spindles in conventional mitotic stages. The peak expression of PfCAP-H is at the metaphase plate. Scale bars = 2 µm. (**B**) Example of schizont exhibiting two mitotic spindles within one dividing nuclei and thus suggesting the existence of schizogony with limited karyokinesis during blood stages. Scale bars = 2 µm; the image is projection of 20 z-slices.

For higher resolution imaging, we utilized ultrastructure-expansion microscopy (U-ExM) to more precisely localize PfCAP-H and examine its role during cell division in these parasites. For these studies, we have included a fluorophore conjugated to N-hydroxysuccinimide (NHS) (herein referred to as “NHS-Ester”) as a non-specific stain for protein density (30), SYTOX Deep Red as a DNA stain, α-V5 for smV5-tagged PfCAP-H, and α-tubulin for mitotic spindles. As noted above, *P. falciparum* undergoes an atypical cell division known as schizogony, where individual nuclei undergo asynchronous S/M cycles, followed by a final semi-synchronous cycle coupled with the budding of the daughter cells (4, 29). Given the complexity of schizogony and decondensed chromosomes in *Plasmodium falciparum*, it is challenging to distinctly visualize different stages of mitosis in these parasites. Therefore, we relied upon the positioning of mitotic apparatus/microtubule organizing centers and microtubules to evaluate the distinct phases of mitosis in these parasites. As shown in the Fig. 2A, in nuclei undergoing mitosis, we observed that PfCAP-H shows a speckled pattern at the plus end tip of the mitotic spindles. The signal intensifies and organizes as clusters at the metaphase plate where chromosomes are typically aligned during metaphase. Subsequently, these signals resume their speckled pattern at the tip of mitotic spindles during anaphase and telophase, which finally becomes more diffuse at the end of the cell cycle. To further confirm the localization of PfCAP-H to the plus end of mitotic spindles, we generated a parasite strain where the kinetochore marker, PfNDC80/PF3D7_0616200 (31, 32), was additionally epitope tagged at its endogenous locus with spaghetti monster HA (smHA) (Fig. S4A B). Immunofluorescence of these dual-tagged parasites demonstrated that PfCAP-H localizes within the surrounding PfNDC80 staining, suggesting that PfCAP-H is on the chromosomal side (Fig. S4C). These results demonstrate that PfCAP-H shows a dynamic pattern during different stages of mitosis and provides a marker for the metaphase plate in mitotically active parasites.

Interestingly, we observed that, in ~10% of schizonts (3 of 31), two duplicated hemi-spindle and/or mitotic spindles are present within a single nucleus (Fig. 2B). These unusual events suggest that these schizonts follow unconventional schizogony where already duplicated genomes with a single, undivided nucleus have already started the next round of mitosis before completing karyokinesis. This supports a model of schizogony with limited karyokinesis (4) occurring at times during asexual development.

### PfCAP-H is a member of the condensin I complex in *Plasmodium falciparum*

**Fig 3:**
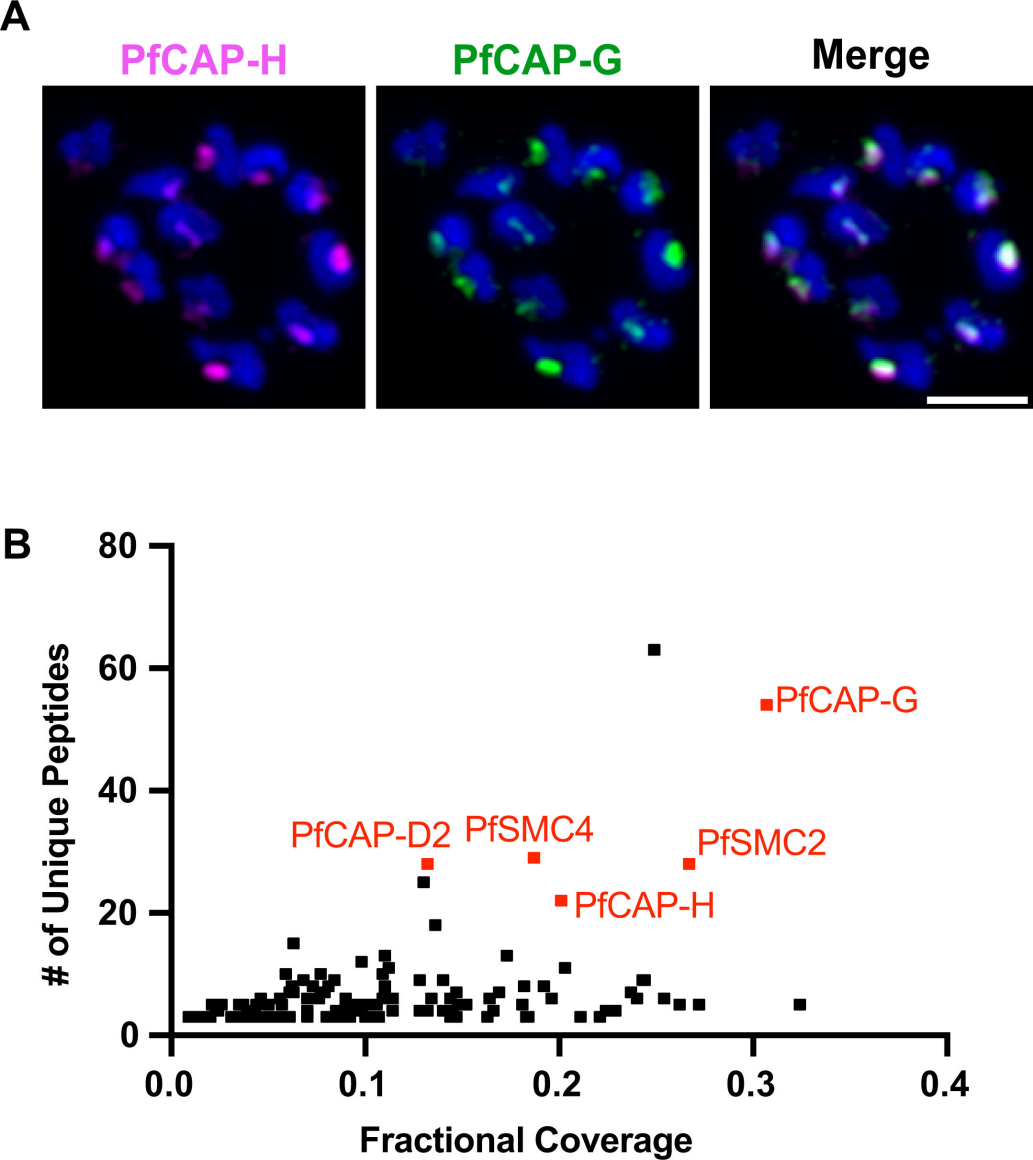
PfCAP-H is a member of the condensin I complex. (**A**) Schizonts from PfCAP-H^DiCre^/PfCAP-G parasites were fixed and stained with α-V5 against PfCAP-H and α-HA against PfCAP-G (*n* = 3). The slide-based IFA showed that PfCAP-H colocalizes with PfCAP-G. Scale bars = 2 µm. (**B**) Proximity labeling in PfCAP-G^BirA^ parasites demonstrates that PfCAP-H interacts with PfCAP-G and other members of the condensin I complex (representative example shown from one of three biological replicates).

The observed expression and localization pattern of PfCAP-H resembles the pattern exhibited by SMC core members (PbSMC2/4) and PfCAP-G of the condensin I complex in *Plasmodium* spp. (14, 16), suggesting that PfCAP-H is a member of the condensin I complex in these parasites. To confirm this hypothesis in *Plasmodium falciparum*, we performed two independent experiments. First, we investigated whether PfCAP-H interacts with PfCAP-G, a non-SMC member of the condensin I complex, by performing IFA with PfCAP-H^DiCre^ /PfCAP-G parasites, where the endogenous PfCAP-G has a smHA epitope in the smV5-tagged PfCAP-H strain. The dual-transgenic line was confirmed by integration PCR and whole genome sequencing (Fig. S5C, sequence reads deposited in NCBI Sequence Read Archive, PRJNA1091348). PfCAP-H and PfCAP-G colocalize with each other and displayed a similar dynamic expression pattern throughout schizogony (Fig. 3A; Fig. S5D), suggesting that these two proteins interact with each other throughout this stage. Second, we used an alternative approach where we fused a promiscuous version of the biotin ligase BirA (33) to PfCAP-G in 3D7 parasites to generate a new transgenic PfCAP-G^BirA^ strain. We used these PfCAP-G^BirA^ parasites to identify the proteins that interact with PfCAP-G using BioID-based proximity labeling in late schizonts coupled with mass spectrometry (Fig. 3B; Table S1) (33). As anticipated, all the members of the condensin I complex, including PfCAP-H, were among the top hits, implying that PfCAP-G is a true partner of PfCAP-H along with other members of the condensin I complex. Altogether, these data establish PfCAP-H as a *bona fide* member of the condensin I complex in *P. falciparum*.

### PfCAP-H is essential for asexual development of blood stage parasites

**Fig 4:**
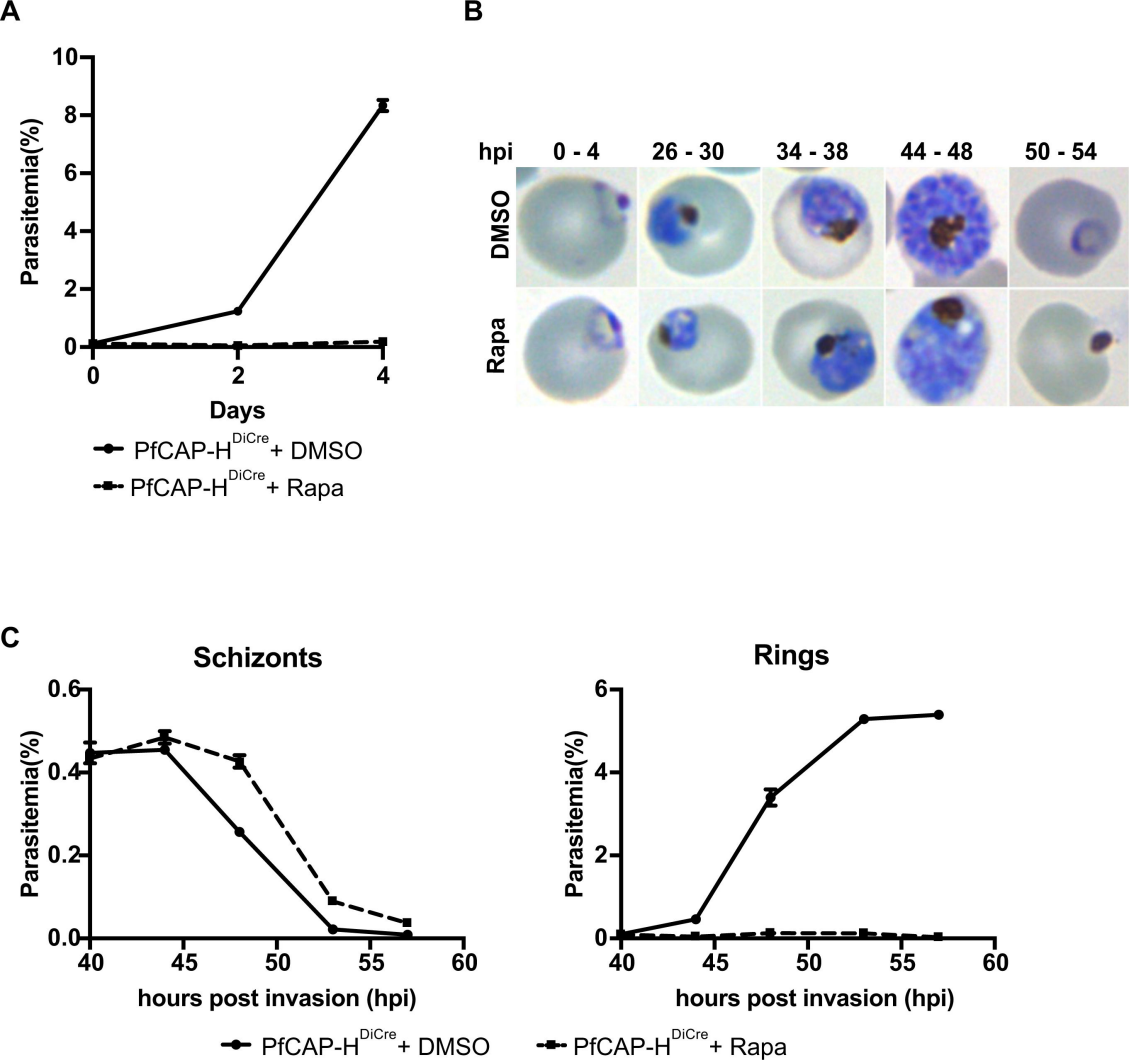
PfCAP-H is essential for the asexual development of parasites. (**A**) PfCAP-H^DiCre^ parasites were treated with rapamycin, and their growth was monitored over two consecutive cycles by flow cytometry. Loss of PfCAP-H showed a lethal effect on parasite asexual growth development. Error bars show the standard deviation from mean of three independent biological replicates. (**B**) The Hemacolor-stained smear was prepared for the first growth cycle to observe the progression of asexual stages. In rapamycin-treated parasites, we observed that the parasites are arrested at the late blood stages, and no new invasion of red blood cells was observed. (**C**) The time course of PfCAP-H parasites cultured with rapamycin/DMSO was collected every 5 hours from 40 to 60 hours post-invasion (hpi), and parasitemia was measured by flow cytometry (*n* = 3, error = SEM) to examine the duration of egress and invasion. Rapamycin-treated parasites did not show any significant difference in the progression of growth; however, they could not invade new red blood cells.

The condensin I complex plays a crucial role during cell division. Knockdown of PfCAP-G produces a significant fitness defect in *P. falciparum*, and PbSMC2/4 could not be knocked out in *P. berghei* asexual parasites (14, 16). To directly determine the consequences of complete removal of PfCAP-H, we performed a replication growth assay where we treated synchronized early ring (0–4 hours) staged PfCAP-H*^DiCre^* parasites with rapamycin or DMSO and monitored their growth for two consecutive cycles using flow cytometry. In these parasites (Fig. 1A), rapamycin dimerization of the DiCre subunits mediates excision of the PfCAP-H gene flanked by two *loxP* sites, resulting in a PfCAP-H knockout (PfCAP-H iKO) (28). In PfCAP-H iKO parasites, whole locus PCR amplification detected 4.8 kb truncated gene locus in rapamycin-treated parasites compared with full-length 9.5 kb modified PfCAP-H locus in DMSO-treated parasites (Fig. S2A). Furthermore, IFA demonstrated that the rapamycin treatment resulted in a complete loss of PfCAP-H protein in PfCAP-H iKO parasites (Fig. S2B). We used the parental 3D7*pfs47^DiCre^* parasite strain as a control. The rapamycin-treated *PfCAP-H^DiCre^* parasites exhibited a 97.8% ± 0.6% growth defect, resulting in death of parasites within the same cycle of treatment when compared with DMSO-treated parasites (Fig. 4A).

To evaluate the growth phenotype in more detail, we examined field-stained parasites to determine the developmental stage of the arrested parasites. At this level of resolution, we observed that PfCAP-H iKO parasites grew normally until the early schizont stage [approximately 30 hpi. After this point, parasites had abnormal morphology and, eventually, failed to generate newly invaded rings (Fig. 4B). Because we did not observe an accumulation of unruptured schizonts, we measured the timing of egress (and potential reinvasion or lack thereof), in the presence and absence of PfCAP-H knockout, by flow cytometry. Notably, we did not find any significant difference in the timing of schizont egress in the absence of PfCAP-H. PfCAP-H iKO parasites ruptured at the same time as control parasites, suggesting that the loss of PfCAP-H does not cause an egress defect. However, the egressed PfCAP-H iKO parasites did not form new rings (Fig. 4C).

We next asked whether this growth defect is due to alterations in DNA replication in PfCAP-H iKO parasites. To address this, we measured the DNA content of rapamycin or DMSO-treated parasites at 40 hpi, when parasites are at the peak of their dividing state. Parasite DNA was labeled with SYBR Green and quantified by the measurement of mean fluorescence intensity by flow cytometry. Remarkably, we did not find any significant difference in the total DNA content in PfCAP-H iKO and control parasites (Fig. S6), indicating that deletion of PfCAP-H does not have a major effect on DNA replication in the knockout parasites.

### PfCAP-H depletion does not affect the centrosome and mitotic spindle formation

**Fig 5:**
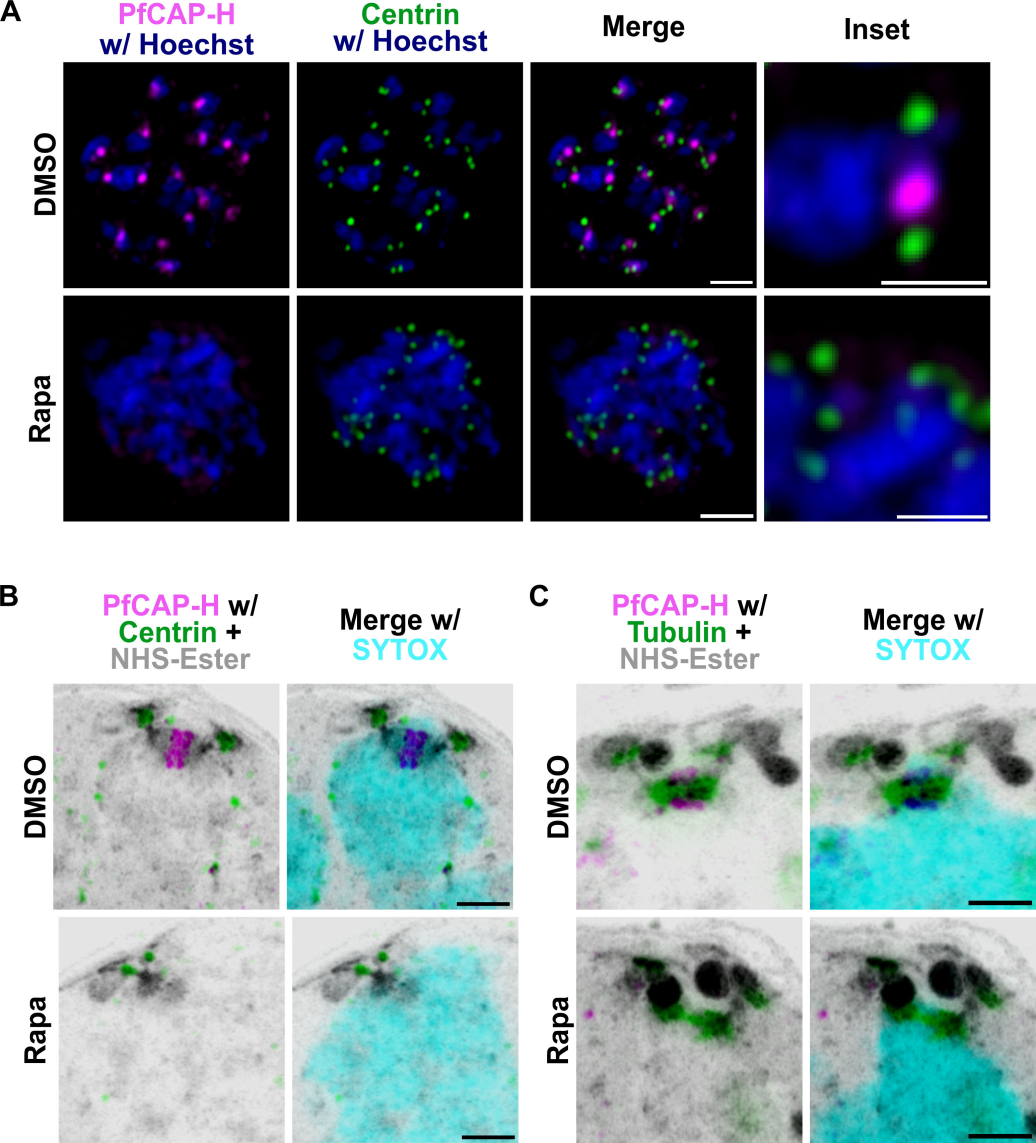
PfCAP-H is dispensable for centrosome dynamics and microtubule formation. PfCAP-H^DiCre^/PfCAP-G parasites were synchronized, treated with rapamycin/DMSO, and collected for slide-based IFA and U-ExM. The samples were stained with α-V5 (PfCAP-H, magenta), α-Centrin (Centrosome marker, green), and α-tubulin (green). In addition, for U-ExM, NHS-Ester (grayscale) and SYTOX (cyan) were used. (**A**) In PfCAP-H-deficient parasites, the slide-based IFA demonstrated that the centrosomes are duplicated but their separation is affected. The highly resolved U-ExM further confirms the similar observation. The U-ExM showed that centrosome (**B**) and microtubule formation (**C**) look similar in both the conditions. Scale bar = 2 µm for images and inset.

Centrosomes act as the microtubule-organizing center, and failure to duplicate or separate could lead to aberrant cell division (34). We interrogated whether the depletion of PfCAP-H affects the biogenesis and dynamics of centrosomes during mitosis with U-ExM on the DMSO and rapamycin-treated PfCAP-H^DiCre^ strains probed with α-V5, α-Centrin, NHS-Ester, and SYTOX (35). Surprisingly, the centrosomes are present and duplicated normally, even without PfCAP-H (Fig. 5A and B). However, in the absence of PfCAP-H, the centrosomes remain clustered over the mass of unsegmented nuclei. These results suggest that the phenotype observed by depletion of PfCAP-H is not due to failure in centrosome duplication or separation but to some other defect.

We monitored if mitotic spindles could form in the absence of PfCAP-H by direct visualization of these structures by U-ExM with *α*-tubulin in dividing parasites. In rapamycin-treated parasites, we observed normal mitotic spindle formation, suggesting that the PfCAP-H is also not required for mitotic spindle assembly (Fig. 5C). Taken together, these results imply that the PfCAP-H function is dispensable for centrosome and mitotic spindle assembly.

### Loss of PfCAP-H deters the proper localization of PfCAP-G on mitotic chromatin

**Fig 6:**
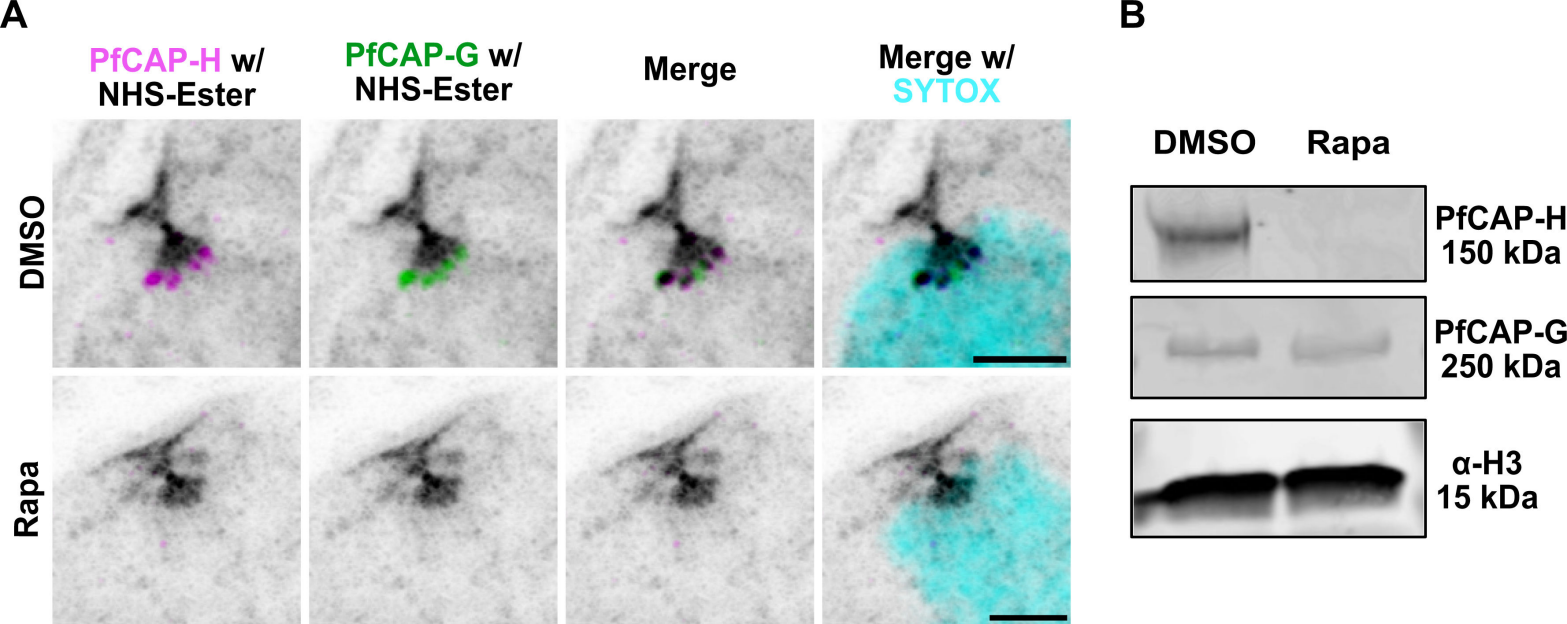
PfCAP-H is essential for assembly of the condensin I complex. PfCAP-H^DiCre^/PfCAP-G parasites were synchronized, treated with rapamycin/DMSO, and collected for U-ExM. The samples were stained with α-V5 (PfCAP-H, magenta), α-HA (PfCAP-G, green), NHS-Ester (grayscale), and SYTOX (cyan). (**A**) In PfCAP-H-deficient parasites, the highly resolved U-ExM showed that localization of PfCAP-G to the mitotic chromosome is lost in the absence of PfCAP-H. (**B**) The immunoblot analysis displayed that, in PfCAP-H KO parasites, expression of PfCAP-G is unaffected. Scale bar = 2 µm.

PfCAP-H homologs play a vital role in maintaining the ring-like structure of condensin I complex and ensuring their collective function as chromatin condensers in other eukaryotes (17). To investigate if this function was preserved for PfCAP-H, we evaluated the expression and localization of PfCAP-G in the presence or absence of PfCAP-H. With U-ExM, we found that the PfCAP-G is no longer localized to the mitotic chromosomes in the PfCAP-H iKO parasites (Fig. 6A). However, the expression of PfCAP-G is not affected in the absence of PfCAP-H (Fig. 6B). This observation suggests that the depletion of PfCAP-H does not affect the expression of PfCAP-G, but it is crucial for the loading of PfCAP-G on the mitotic chromosomes. Thus, the data imply that PfCAP-H is critical for the assembly of non-SMC members of the condensin I complex in *Plasmodium falciparum*.

### Depletion of PfCAP-H affects karyokinesis but does not impede cytokinesis

**Fig 7:**
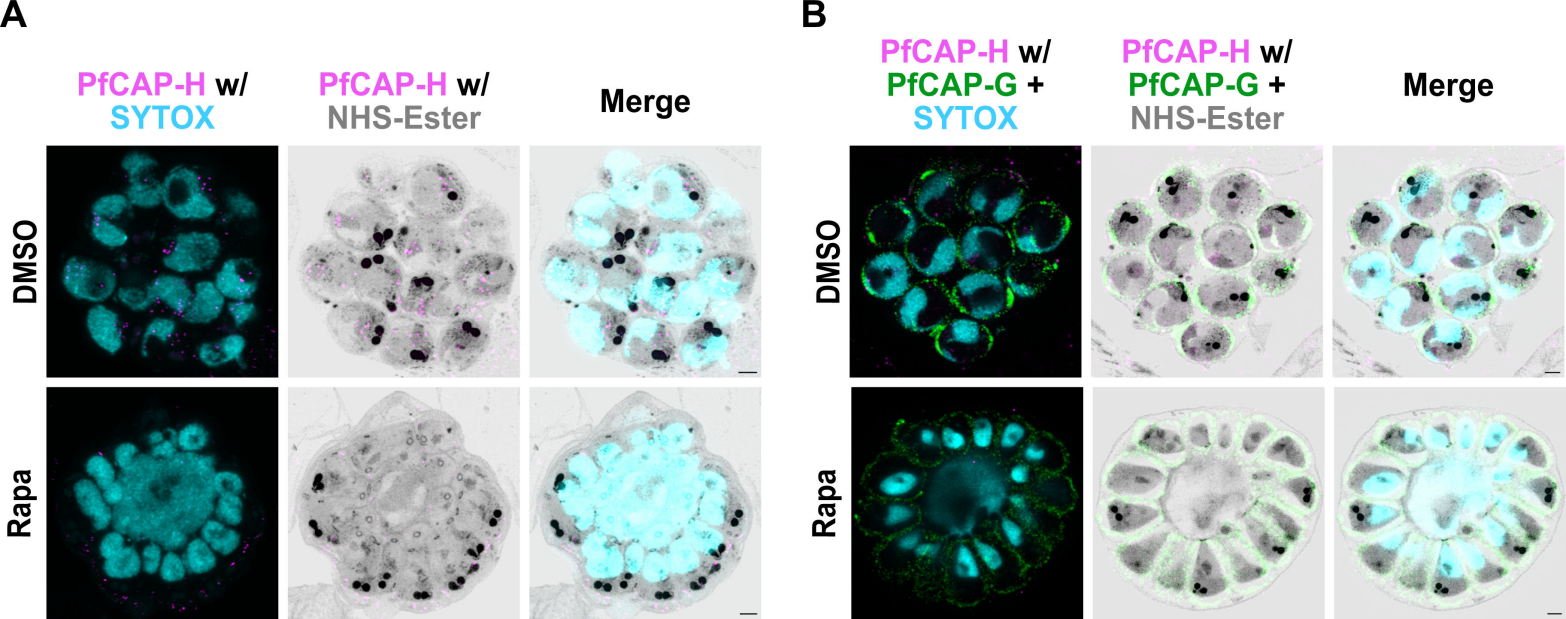
PfCAP-H knockout affects karyokinesis but not cytokinesis. Synchronized PfCAP-H^DiCre^ parasites were treated with rapamycin or DMSO and probed with α-V5 (PfCAP-H, magenta), α-PfGAP45 [inner membrane complex (IMC) green], NHS-Ester (protein, greyscale), and SYTOX (nucleus, cyan) for U-ExM (*n* = 3). (**A and B**) E64-arrested late matured schizonts; PfCAP-H KO parasites showed a giant agglomerate of incompletely separated nuclei while the IMC appeared to be surrounding all potential merozoites, implying unaffected cytokinesis. Scale bar = 2 µm.

Our observations thus far suggest that the defect in PfCAP-H iKO parasites is due to abnormal nuclear division rather than DNA replication. We thus interrogated the process of karyokinesis in these parasites during their asexual development. Again, we utilized U-ExM along with NHS-Ester, α-V5 for PfCAP-H, and SYTOX to visualize the parasite DNA. At 40 hpi, we observed that PfCAP-H iKO parasites showed defective nuclear division. At the same stage, the control parasites had normal nuclear division (Fig. S7). We further followed the karyokinesis until the end of one development cycle by trapping parasites prior to egress with the cysteine protease inhibitor E64 (36). Strikingly, we observed that fully matured PfCAP-H iKO parasites showed a large agglomerate of incompletely separated nuclear material compared with the >20 normally separated nuclei in control parasites (Fig. 7A). This result suggests that the defect of chromosome segregation leads to a defect in karyokinesis.

To evaluate cytokinesis, E64-treated parasites were stained with antibodies against PfCAP-H (V5) and PfGAP45, an IMC-associated protein (37). With U-ExM, we demonstrated that despite abnormal karyokinesis, the IMC still surrounds the nascent merozoites. A varied amount of nuclear material is contained in the forming merozoites, with some nuclear material observed in streaks within the contracted basal complex as well as additional nuclear material in the residual body (Fig. 7B). While there may be abnormalities in the final steps of abscission, these results suggest that the processes of cytokinesis, including IMC formation and basal complex contraction, are largely intact. These schizonts still egress (Fig. 4C) but do not form new ring-stage parasites.

### PfCAP-H is likely dispensable for sexual stage development

**Fig 8:**
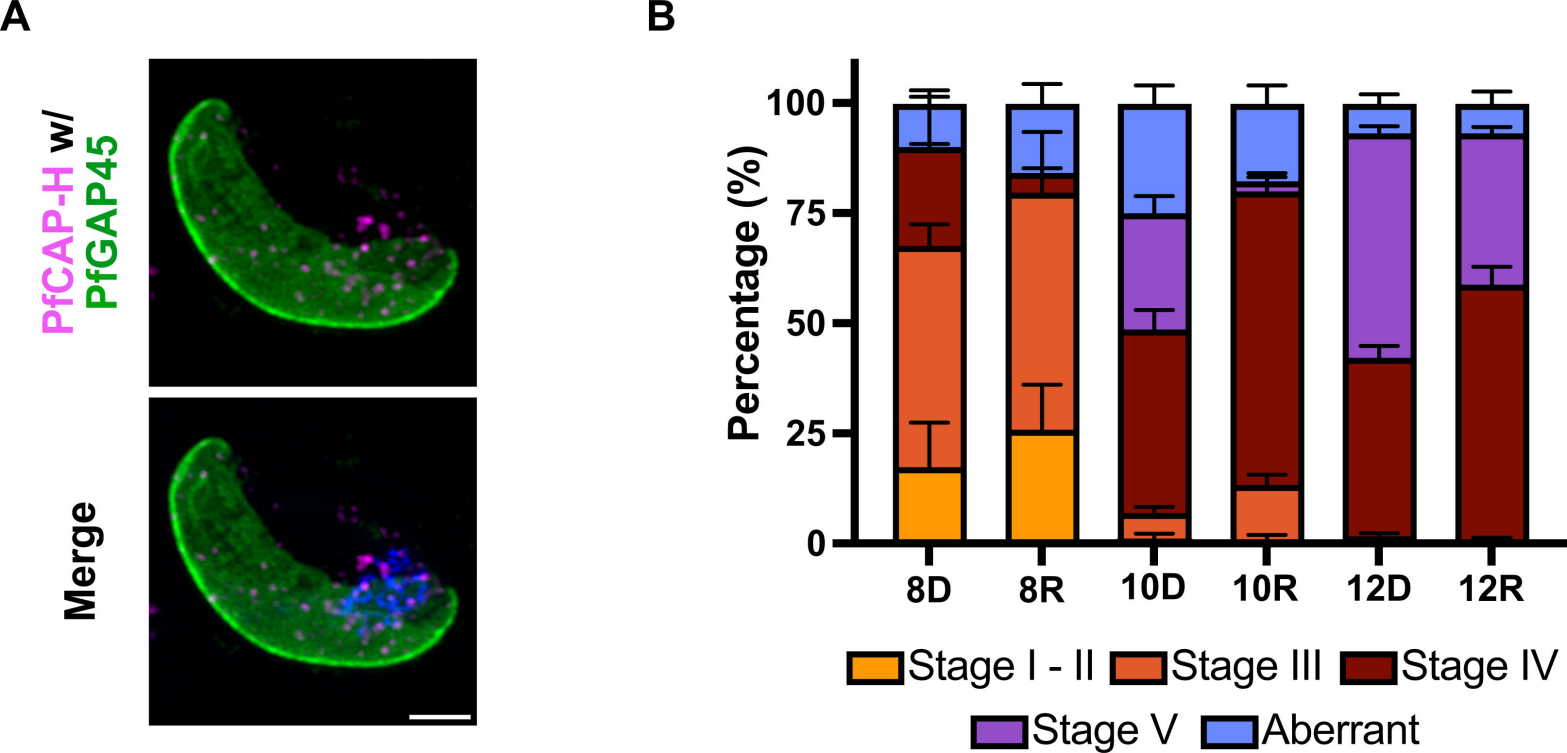
PfCAP-H is likely dispensable for gametogenesis. (**A**) Slide-based IFA was performed for the localization of PfCAP-H in sexual stages (Stage III) on day 8 post-induction. The slides were probed with α-V5 (magenta) for PfCAP-H and α-PfGAP-45 as IMC markers (green). IFA showed that PfCAP-H is present, adjacent to the nucleus in stage III gametocytes. Scale bar = 2 µm. (**B**) The progression of gametogenesis was examined in PfCAP-H^DiCre^ parasites and treated with rapamycin or DMSO, from day 8 to Day 12 post-induction, by calculating different stages (Stage I to Stage V) with Hemacolor-stained thin smears. The aberrant category includes parasites that appear stressed, distorted, and abnormal parasites. In contrast, the unknown category comprises the gametocytes, which were difficult to categorize based on standard categorization in reference (38). The experiment was performed in two independent biological replicates. The error bar indicates SEM calculated by GraphPad Prism.

Given that PbSMC2/4 and PfCAP-G are necessary for the sexual development of these parasites, we asked whether PfCAP-H is required for the sexual development of *Plasmodium falciparum*. To investigate the role of PfCAP-H in gametocyte development, we induced sexual commitment in synchronized PfCAP-H^DiCre^ parasites, treated with rapamycin or DMSO, and then monitored the gametocytogenesis over 12 days. The gametocyte conversion rate was similar in both PfCAP-H iKO and control parasites. In addition, the absolute gametocytemia on day 6 after induction was similar at 1.9 ± 0.3% and 2.0 ± 0.1% (mean ± SD) in the DMSO and rapamycin conditions, respectively. In stage III gametocytes, PfCAP-H localizes adjacent to the nucleus (Fig. 8A). Intriguingly, we did not find any significant morphological difference between PfCAP-H iKO and control parasites throughout the 12 days of the assay (Fig. 8B, Fig. S8A and B).

## DISCUSSION

To unravel the function of PfCAP-H during the erythrocytic development in *Plasmodium falciparum*, we employed an inducible knockout parasite strain and ultrastructure expansion microscopy. We revealed that PfCAP-H is essential for asexual blood stages. The PfCAP-H knockout exhibited robust nuclear division defect, while the cytokinesis continued relatively normally. However, this is distinct from most eukaryotes, where the surveillance system verifies that nuclear division is completed typically before the onset of cytokinesis (39, 40). Thus, this work corroborates previous studies that showed that karyokinesis and cytokinesis are independent and these malarial parasites lack some aspects of the surveillance systems present in other eukaryotes (29, 41, 42).

In many eukaryotes, condensation of chromosomes during mitosis is a crucial step to ensure the faithful division of the genomic material in daughter cells. However, it is intriguing to comprehend how this equal distribution of genome is maintained and regulated in lower eukaryotes that possess decondensed forms of chromosomes throughout their life cycle (43). Remarkably, despite the absence of condensed chromosomes, these lower eukaryotes have retained the highly conserved condensin complexes, which facilitate the condensation of chromosomes (11). This forces us to ponder the significance of these complexes in such an unusual scenario. So far, the function of condensin complexes in *Saccharomyces cerevisiae* has been extensively studied to decipher the equal distribution of the genome with such atypical decondensed chromosomes during closed mitosis (27, 44–46). These complexes are recently identified in *Plasmodium* spp., which share decondensed chromosomes similar to fission yeast (2, 14, 16). However, attributing to their small nuclear size (~1 µm diameter) and underexplored molecular mediators during mitosis compared with model organisms (29), it is challenging to investigate the chromosome dynamics in *Plasmodium* spp. during mitosis. The foremost step in the quest of interrogating chromosome dynamics is to get detailed evidence of different mitotic stages in these parasites. Strikingly, the recent establishment of ultrastructure expansion microscopy techniques in these parasites has unlocked the feasibility of studying the nuclear-related processes in these parasites (47). Leveraging U-ExM capability to expand the size of parasites, we captured highly resolved images of mitotic events in these parasites. Furthermore, we showed that the PfCAP-H is highly expressed at the metaphase plate and propose that PfCAP-H could be used as a marker for the metaphase plate to mark the mitotically active nucleus during schizogony. Consequently, subcellular localization of PfCAP-H at the metaphase plate, along with the cues from localization of the mitotic spindle, as in the yeast mitosis model (44), would further aid in advancing our knowledge on mitosis in malarial parasites.

This study demonstrates that PfCAP-H interacts with PfCAP-G and is a crucial member of the condensin I complex. The U-ExM revealed that PfCAP-G does not localize to the mitotic chromosome in the absence of PfCAP-H, suggesting that PfCAP-H is required to load PfCAP-G on the mitotic chromosome, similar to its homologs in *Drosophila* and humans (18, 26). Furthermore, the sequence analysis of PfCAP-H showed that PfCAP-H contains highly conserved N- and C-terminal regions, which are the sites of interaction for SMC2/4 in its homologs (11, 21), inferring that PfCAP-H might also bind to SMC2/4 via these highly conserved regions. In *P. berghei*, it was shown that PbCAP-H co-immunoprecipitates with SMC2/4 in late schizonts but not in early schizonts (14). This information hints that SMC2/4 can bind to chromosomes alone; however, PfCAP-H is required to assemble the subunits to function as a condensin I holo-complex. We propose that PfCAP-H plays a vital role in the assembly of the condensin I complex on the mitotic chromosomes in *Plasmodium* parasites, comparable to its homolog in other systems (17). Furthermore, the essentiality of this complex suggests that chromosome condensation does occur in these parasites during mitosis, albeit to a lesser extent than in higher eukaryotes.

PfCAP-H is dynamically localized during schizogony with peak expression during its growth phase, followed by diffuse localization at the end of the schizogony. This expression and localization resemble the similar pattern displayed by the condensin I complex in several other eukaryotes during mitosis (9). In model organisms, condensin complex localization and activity are tightly regulated through phosphorylation by mitotic kinases (48). For example, the mitotic kinase cdc2-cyclin B phosphorylates the non-SMC subunits of condensin in *Xenopus* and human cells to condense the chromosome (49). Barren, the *Drosophila* homolog of PfCAP-H, recruitment to mitotic chromosomes is facilitated by phosphorylation by Aurora B kinase (50). Notably, phosphoproteomic analysis in *Plasmodium falciparum* showed that all the subunits of the condensin I complex, including PfCAP-H, are phosphorylated during asexual blood stages (6, 51). We thus speculate that the condensin I complex is likely regulated by phosphorylation in these parasites. However, further study is required to investigate the mechanisms for controlling condensin I activity in *Plasmodium falciparum*.

Interestingly, while scrutinizing the localization of PfCAP-H in blood stages, we observed that some schizonts exhibited unconventional schizogony where already duplicated nuclei restart the next round of mitosis before completing karyokinesis. This observation agrees with previous studies of asexual blood stage development of *P. falciparum* (7, 52). This variation in schizogony has been observed in the mosquito midgut during sporozoite formation of *Plasmodium* spp. (53) and is described as schizogony with limited karyokinesis (4). Conversely, the evidence of such variation in the asexual blood stage suggests that schizogony is somewhat fluid and is not restricted only to classic schizogony. However, the factors responsible for such flexibility in blood stages remain to be explored.

**Fig 9:**
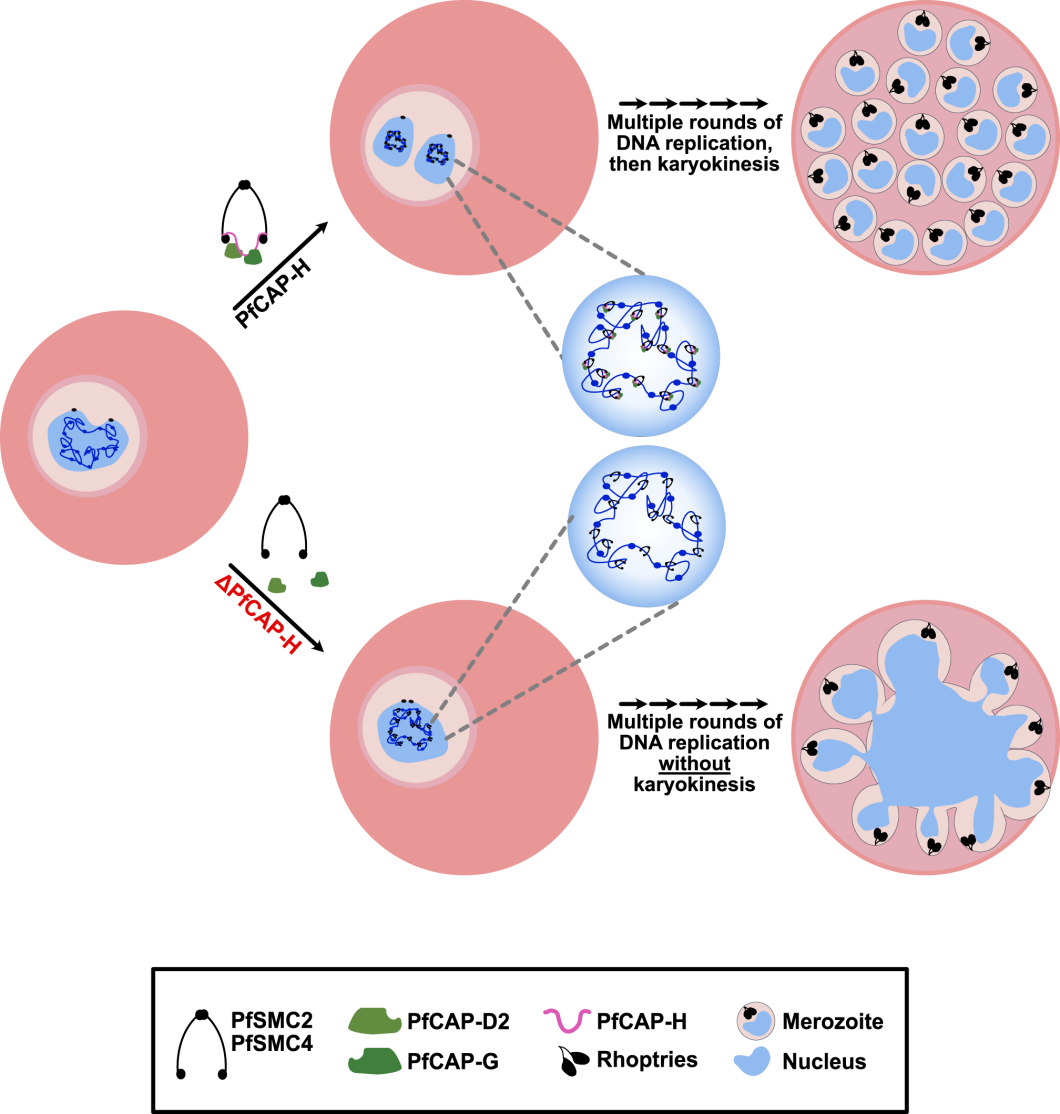
Proposed model of PfCAP-H function during blood stage development. Schematic representation of the proposed mechanism by which PfCAP-H functions during schizogony. PfCAP-H plays a pivotal role in facilitating the formation of condensin I complex on the mitotic chromosomes. In parasites lacking PfCAP-H, the process of karyokinesis is significantly impaired, while the cytokinesis remains unaffected.

In summary, we have shown that PfCAP-H is essential for the asexual blood stage development. PfCAP-H is critical for assembling the condensin I complex on the mitotic chromosomes. The knockout of PfCAP-H results in agglomeration of nuclei, possibly due to improper chromosome segregation, and defective karyokinesis—while cytokinesis remains largely normal (Fig. 9). Furthermore, PfCAP-H is not required for centrosome duplication or mitotic spindle assembly during schizogony. Overall, this study sheds light on the function of the condensin I complex during mitosis in these parasites and emphasizes their importance in lowering eukaryotes with primitive mitotic features of decondensed chromosomes.

## MATERIALS AND METHODS

### Plasmid construction

#### pPG25 (for smV5-tagged PfCAP-H)

The 5′ homology region (HR) and 3′ HR region were amplified from Pf3D7 genomic DNA with oJDD6763/6764 and oJDD6766/6767, respectively. The codon-altered PfCAP-H gene blocks (GB70.2 and GB71, IDT DNA) were amplified with oJDD6598/6599 and oJDD6196/6197, respectively. The smV5 and hDHFR region was amplified from pPG12 with oJDD6765/6086. The pGEM backbone for this plasmid was amplified from pPG12 with oJDD6083/6084. The six pieces were assembled with the Golden Gate *BsaI*-HF v2 Assembly Kit (NEB). Three guide RNA (gRNA) plasmids were used to target the PfCAP-H locus. The guides were annealed and ligated into *BpiI*-digested pRR216 plasmid (SpCas9 expression plasmid) to construct pJPM59 (oJDD4921/4922), pCJM57 (oJDD5791/oJDD5792), and pCJM58 (oJDD5793/oJDD5794). The *EcoRI/NotI* linearized pPG25 plasmid was co-transfected with the three guide RNA-containing plasmids into 3D7*pfs47*DiCre parasites to generate PfCAP-H^DiCre^ parasites.

#### pRR85 (for generating BirA fused PfCAP-G)

The 5′ HR and 3′ HR regions were amplified from 3D7 genomic DNA with primers oJDD3881/3882 and oJDD3875/3876. Codon-altered PfCAP-G was amplified from gene block GB04 with oJDD3883/3884. The U6 promoter, gRNA for PfCAP-G, and U6 terminator were amplified from pBAM203 which were assembled by overlapping PCR extension with oJDD3877/3878 and oJDD3879/3880. These five pieces were assembled by overlapping PCR and ligated into pRR28 cut with *NotI* and *XhoI*. The *SpeI*-linearized pRR85 plasmid was co-transfected with a SpCas9 expression plasmid into the 3D7 parasite strain to generate PfCAP-G^BirA^ parasites.

#### pCJM22 (for generating smHA-tagged PfCAP-G)

The PfCAP-G region was digested from pRR85 with *NotI/XhoI*. The smHA and BSD sequences were obtained from pCJM01 by *NotI/XhoI* digestion. These two digested fragments were ligated with T4 DNA ligase to get pCJM22. For transfection, the Spe1-linearized pCJM22 plasmid was transfected with the SpCas9 expression plasmid in the PfCAP-H^DiCre^ strain to generate PfCAP-H^DiCre^/PfCAP-G parasites.

#### pPG89 (for generating smHA-tagged PfNDC80)

The PfNDC80 region was PCR amplified with primers oJDD8427/8430 and digested with *NotI/XhoI*. The smHA and BSD sequences were obtained from pCJM22 by *NotI/XhoI* digestion. These two digested fragments were ligated with T4 DNA ligase to get pPG89. For transfection, the Spe1-linearized pPG89 plasmid was transfected with three guide RNA-containing plasmids in the PfCAP-H^DiCre^ strain to generate PfCAP-H^DiCre^/PfNDC80 parasites. All primers and geneblocks are shown in Table S2.

### Reagents and antibodies

All the primers used in this study were purchased from Thermo Fisher, gene blocks were purchased from IDT DNA, and restriction enzymes were purchased from New England BioLabs. The primary antibodies used in this study are the following: mouse *α*-V5 (clone SV5-Pk2, Bio-Rad); rabbit *α*-V5 (ICL, RV5-45A-Z); rat *α*-hemagglutinin (HA, clone 3F10, Sigma); mouse *α*-tubulin (clone B-5-1-2, Sigma); rabbit *α*-Histone H3 (ab1971, Abcam); mouse *α*-Centrin (CrCen clone 20H5, EMD Millipore); and rabbit *α*-PfGAP45 [a gift from Julian Rayner at the University of Cambridge (54)]. Secondary antibodies and other reagents (Alexa Fluor 405 NHS-Ester, SYTOX Deep Red Nucleic Acid Stain, and Hoechst 33342 Solution) used for microscopy were purchased from Thermo Fisher.

### Sequence alignments

The FASTA sequence of *Plasmodium falciparum* PfCAP-H (PF3D7_1304000) and its orthologs including *Plasmodium berghei* PbCAP-H (PBANKA_1402500), *Saccharomyces cerevisiae* BRN1 (P38170), *Schizosaccharomyces pombe* CND2 (Q9Y7R3), *Arabidopsis thaliana* CAPH (Q564K3), *Xenopus laevis* NCAP-H (O13067), *Homo sapiens* NCAP-H (Q15003), and *Drosophila melanogaster* Barren (P91663) were obtained from UniProt or PlasmoDB. The sequence identity and similarity were calculated using EMBOSS Needle (55). Multiple sequence alignment was carried out using the default MUSCLE algorithm with MEGA software, and aligned sequences were analyzed using ESPript 3.0 (56).

### *Plasmodium falciparum* culture and transfection

The *Plasmodium falciparum* 3D7 strain was obtained from the Walter and Elizabeth Hall Institute, and the 3D7*pfs47*DiCre parasite strain was obtained from Dr. Ellen Knuepfer (55). Parasites were cultured in human O+ erythrocytes (purchased from commercial vendor) at 4% hematocrit (HCT) in RPMI-1640 (Sigma) supplemented with 25 mM HEPES [4-(2-hydroxyethel)-1-piperazineethanesulfonic acid] (EMD Biosciences), 0.21% sodium bicarbonate (Sigma), 50 mg/L hypoxanthine (Sigma), and 0.5% Albumax II (Invitrogen) and were kept at 37°C with a mixture of gases (5% CO_2_, 1% O_2_, and 95% N_2_). Parasite growth was monitored by staining with Hemacolor (Sigma) staining solutions and observed under the microscope. The cultures were synchronized by combining Percoll and sorbitol treatments (57, 58).

To generate transgenic parasites, 60 µg of donor plasmid was linearized by digestion and co-transfected with 60 µg of SpCas9-plasmids containing gRNA into 10% ring-staged *P. falciparum* (3D7*pfs47*DiCre or 3D7) parasites by electroporation. The electroporation was performed at settings of 310 V, 950 µF, and infinite Ω in a 0.2-cm cuvette with Bio-Rad Gene Pulser. The parasites were cultured and selected with appropriate drugs. The PfCAP-H^DiCre^ and PfCAP-G^BirA^ parasites were selected with 2.5 nM WR99210 (Jacobus Pharmaceuticals), and PfCAP-H^DiCre^/CAP-G parasites were selected with 2.5 μg/mL blasticidin (Research Products International). Single clones of these transfected parasites were obtained by limitation dilution. The transgenic parasites were verified by PCR amplification using primers and/or whole genome sequencing of harvested genomic DNA (Biobasic Blood Genomic DNA Miniprep Kit). To induce excision of the PfCAP-H gene, 0–4-hour young parasites were treated with 100 nM rapamycin for up to 12 hours, followed by washing with new culture media (28).

### Gametocyte induction

Trophozoite-stage parasites at 3% parasitemia and 2% HCT were cultured with a 50% conditioned AlbuMax II medium. After 2 days, the culture was treated with 0.25 mg/mL heparin (Alpha Aesar A16198) and 2.5 mM N-acetylglucosamine to prevent subsequent invasion of asexual-stage parasites. Gametocyte conversion and morphology were evaluated by blood smear stained with Hemacolor staining solution under light microscopy. The experiments were performed in two independent biological replicates. The gametocyte conversion was calculated by the ratio of gametocytes on day 6 to ring parasitemia on day 2. IFAs were performed on day 8 to localize the protein in gametocyte stages. The gametocytes were categorized as described (38).

### Replication assay

Synchronized ring-stage parasites were seeded with an initial parasitemia at 0.25% in 1% HCT, treated with DMSO or 20 nM rapamycin, and cultured for two consecutive cycles. One hundred-microliter culture was collected from each well on three different days (0, 2, and 4) and then washed once with 0.5% bovine serum albumin (BSA) in 1× phosphate-buffered saline (PBS). The samples were resuspended in 100 µL staining solution containing 1:1,000 SYBR Green I (Invitrogen) dilution in 0.5% BSA/PBS and incubated for 20 mins at room temperature (RT). The stained samples were washed with 0.5% BSA/PBS and then resuspended in 1× PBS. The number of infected red blood cells was determined by flow cytometry (FACSCalibur) using the CellQuest Pro program. The data from 100,000 cells were analyzed by FlowJo X and GraphPad Prism 9 software and represented as mean and SD of triplicates.

### Immunofluorescence assays

For IFA, parasites were smeared onto slides and air dried. The entire process of IFA was done in a humid chamber. The parasites were fixed with 4% (vol/vol) paraformaldehyde (PFA) in 1× PBS for 10 mins and rinsed quickly with 1× PBS. The fixed parasites were permeabilized with 0.1% Triton X-100 in PBS for 10 mins at RT and then washed three times with 1× PBS for 3 mins. The parasites were blocked with a 3% (wt/vol) BSA in PBS for 1 hour at RT. The smear was stained with respective primary antibodies (mouse *α*-V5 1:500, rat *α*-HA 1:250, mouse *α*-Centrin 1:500, and rabbit *α*-V5 1:500) for 1 hour at RT or overnight at 4°C, followed by washing three times with 1× PBS for 5 mins. The samples were incubated with fluorescently labeled secondary antibodies (1:1,000) for 30–45 mins at RT and washed thrice with 1× PBS for 5 mins to remove excess unbound antibodies. The DNA content of the parasites was stained with Hoechst 33342 (1:5,000) in 1× PBS for 20 mins at RT and then quickly rinsed with 1× PBS. The parasites were mounted in Vectashield Vibrance Antifade Mounting Media (Vector Laboratories Inc., H-1700) with coverslips and stored at 4°C until imaging. The z-stacked Images were acquired on a Zeiss LSM900 microscope with Airyscan 2 with 63× objective and analyzed using FIJI software.

### Ultrastructure Expansion Microscopy

For expansion microscopy, we followed a 4-day protocol. On day 1, synchronized parasites at 4%–5% parasitemia were collected and allowed to settle on a poly-D-lysine-coated coverslip for 20 mins at 37°C. Parasites were fixed with pre-warmed 4% (vol/vol) PFA for 20 mins at 37°C, washed thrice with pre-warmed 1× PBS, and crosslinked with 1.4% formaldehyde and 2% acrylamide solution in 1× PBS overnight at 37°C. On day 2, the gel polymerization was done in a gelation chamber. The parasite-coated coverslips were placed onto the mixture of TEMED/APS/monomer solution, kept on ice for 5 mins, and then incubated for 1 hour at 37°C. Post-incubation, the coverslips plus gel were placed in a 6-well dish with 1 mL denaturation buffer for 15 mins with agitation to displace gel from the coverslip. The detached gel was then incubated with 1.5 mL denaturation buffer in a microcentrifuge tube for 90 mins at 95°C. After cooling off, gels were transferred to a petri-dish containing 25 mL ddH_2_O and incubated for 30 mins at RT. The gels were further incubated with 25 mL ddH_2_O overnight at RT to perform the first round of expansion. On day 3, gels were washed twice with 1× PBS for 15 mins at RT. The gels were blocked with 3% BSA/PBS for 30 mins at RT, followed by staining with respective primary antibodies (mouse *α*-V5 1:250, rat *α*-HA 1:100, mouse *α*-tubulin 1:500, mouse *α*-Centrin 1:250, and rabbit *α*-GAP45 1:2500) in 3% BSA/PBS overnight at 4°C. On day 4, gels were washed thrice with 1× PBS with 0.5% Tween-20 for 10 mins at RT with agitation. Gels were stained with their respective secondary antibodies (1:500), Alexa Fluor 405 NHS-Ester (1:250), and SYTOX Deep Red Dye Nucleic Acid Stain (1:1,000) in 1× PBS for 2 hours 30 mins at RT protected from light. The stained gels were washed thrice with 1× PBS/0.5% Tween-20 for 10 mins at RT and then incubated with ddH_2_O for 30 mins. Water was replaced, and gels were incubated overnight at RT for the second round of expansion. The images were captured on a Zeiss LSM900 microscope with Airyscan 2 and analyzed using FIJI software.

### Immunoblots

Parasites were harvested by 0.02% saponin in PBS with protease inhibitors (PIC) (SigmaFast Protease Inhibitor Cocktail) and boiled in 1× Laemmli buffer supplemented with 1× PIC for 5 mins at 95°C. The protein lysate (equivalent to 10^8^ parasites per lane) was run on a 4%–20% Tris-glycine-sodium dodecyl sulfate gel and transferred to a PVDF membrane. The membrane was blocked with Licor Odyssey blocking buffer for 1 hour at RT. The immunoblot was probed with primary antibodies (*α*-V5 1:1000, *α*-HA 1:1000, and *α*-H3 1:2500), followed by incubation with secondary antibodies (1:1,000) diluted in the Licor Odyssey blocking buffer. The immunoblot was scanned on a Licor Odyssey CLx imager system and quantified using volumetric measurement of fluorescence intensity with LiCor Image Studio 4.0.

### BirA biotin proximity labeling

Synchronized 44–46 hour schizont-stage parasites (PfCAP-G^BirA^ and 3D7) were incubated with 150 µM biotin for 6 hours; the Protein Kinase G inhibitor, compound 1, was added to prevent parasite egress (59). After this incubation, parasites were harvested with 0.05% saponin in 1× PBS and protease inhibitors. Parasite pellets were resuspended in 1 mL of RIPA lysis buffer with PIC [50 mM Tris-HCl, Ph 7.5, 150 mM NaCl, 1% NP-40 (Tergitol), 0.5% sodium deoxycholate, and 0.1% sodium dodecyl sulfate] for 1 hour at RT on the rotator and sonicated with microtip sonicator with 30 s at 20 amplitudes, followed by a 3-min incubation on ice. This cycle was repeated twice. The lysate was spun down for 30 mins at max speed to remove hemozoin and other insoluble debris. The streptavidin-coated beads were washed with RIPA/PIC and incubated with the cleared lysate overnight on the rotator at 4°C. After incubation, beads were sequentially washed with RIPA buffer and wash buffer 1 (2% SDS), wash buffer 2 (50 mM HEPES, pH 7.5, 500 mM NaCl, 1 mM EDTA, 1% Triton-X 100, and 0.1% sodium deoxycholate), wash buffer 3 (10 mM Tris HCl, pH 8.0, 250 mM lithium chloride, 1 mM EDTA, 0.5% NP-40, and 0.5% sodium deoxycholate), and wash buffer 4 (50 mM ammonium bicarbonate in ddH20). The sample was resuspended in 40 µL of wash buffer 4 and stored at −80°C until further processing for mass spectrometry. On-bead digestion, followed by liquid chromatography tandem mass spectrometry and data analysis, was performed at the Harvard Taplin Mass Spectrometry Facility. The results were analyzed by comparing the unique and total peptides between PfCAP-G^BirA^ and 3D7 parasites in three independent biological replicates.

## Data Availability

All data generated throughout this study are incorporated into the manuscript and supplementary files. Whole genome sequencing data have been deposited in the NCBI Sequence Read Archive (PRJNA1091348). Protocols, raw data, or any materials employed in this study are available upon request.

## SUPPLEMENTAL MATERIAL

Supplemental Figures mbio.02850-23-s0001.pdf Figures S1 to S8.

Table S1 mbio.02850-23-s0002.xlsx PfCAP-G BioID data.

Table S2 mbio.02850-23-s0003.xlsx Primers and geneblocks.

ASM does not own the copyrights to Supplemental Material that may be linked to, or accessed through, an article. The authors have granted ASM a non-exclusive, world-wide license to publish the Supplemental Material files. Please contact the corresponding author directly for reuse.

## References

[1] World Health Organization. 2022. World malaria report

[2] Gerald N, Mahajan B, Kumar S. 2011. Mitosis in the human malaria parasite Plasmodium falciparum. Eukaryot Cell 10:474–482. doi:10.1128/EC.00314-10 21317311 PMC3127633

[3] Francia ME, Striepen B. 2014. Striepen B: Cell division in apicomplexan parasites. Nat Rev Microbiol 12:125–136. doi:10.1038/nrmicro3184 24384598

[4] Gubbels M-J, Keroack CD, Dangoudoubiyam S, Worliczek HL, Paul AS, Bauwens C, Elsworth B, Engelberg K, Howe DK, Coppens I, Duraisingh MT. 2020. Fussing about fission: defining variety among mainstream and exotic apicomplexan cell division modes. Front Cell Infect Microbiol 10:269. doi:10.3389/fcimb.2020.00269 32582569 PMC7289922

[5] Klaus S, Binder P, Kim J, Machado M, Funaya C, Schaaf V, Klaschka D, Kudulyte A, Cyrklaff M, Laketa V, Höfer T, Guizetti J, Becker NB, Frischknecht F, Schwarz US, Ganter M. 2022. Asynchronous nuclear cycles in multinucleated Plasmodium falciparum facilitate rapid proliferation. Sci Adv 8:eabj5362. doi:10.1126/sciadv.abj5362 35353560 PMC8967237

[6] Ganter M, Goldberg JM, Dvorin JD, Paulo JA, King JG, Tripathi AK, Paul AS, Yang J, Coppens I, Jiang RHY, Elsworth B, Baker DA, Dinglasan RR, Gygi SP, Duraisingh MT. 2017. Plasmodium falciparum CRK4 directs continuous rounds of DNA replication during schizogony. Nat Microbiol 2:17017. doi:10.1038/nmicrobiol.2017.17 28211852 PMC5328244

[7] Rudlaff RM, Kraemer S, Marshman J, Dvorin JD. 2020. Three-dimensional ultrastructure of Plasmodium falciparum throughout cytokinesis. PLoS Pathog 16:e1008587. doi:10.1371/journal.ppat.1008587 32511279 PMC7302870

[8] Rudlaff RM, Kraemer S, Streva VA, Dvorin JD. 2019. An essential contractile ring protein controls cell division in Plasmodium falciparum. Nat Commun 10:2181. doi:10.1038/s41467-019-10214-z 31097714 PMC6522492

[9] Batty P, Gerlich DW. 2019. Mitotic chromosome mechanics: how cells segregate their genome. Trends Cell Biol 29:717–726. doi:10.1016/j.tcb.2019.05.007 31230958

[10] Paulson JR, Hudson DF, Cisneros-Soberanis F, Earnshaw WC. 2021. Mitotic chromosomes. Semin Cell Dev Biol 117:7–29. doi:10.1016/j.semcdb.2021.03.014 33836947 PMC8406421

[11] Hirano T. 2016. Condensin-based chromosome organization from bacteria to vertebrates. Cell 164:847–857. doi:10.1016/j.cell.2016.01.033 26919425

[12] Hirano T. 2012. Condensins: universal organizers of chromosomes with diverse functions. Genes Dev 26:1659–1678. doi:10.1101/gad.194746.112 22855829 PMC3418584

[13] King TD, Leonard CJ, Cooper JC, Nguyen S, Joyce EF, Phadnis N. 2019. Recurrent losses and rapid evolution of the condensin II complex in insects. Mol Biol Evol 36:2195–2204. doi:10.1093/molbev/msz140 31270536 PMC6759200

[14] Pandey R, Abel S, Boucher M, Wall RJ, Zeeshan M, Rea E, Freville A, Lu XM, Brady D, Daniel E, Stanway RR, Wheatley S, Batugedara G, Hollin T, Bottrill AR, Gupta D, Holder AA, Le Roch KG, Tewari R. 2020. Plasmodium condensin core subunits SMC2/SMC4 mediate atypical mitosis and are essential for parasite proliferation and transmission. Cell Rep 30:1883–1897. doi:10.1016/j.celrep.2020.01.033 32049018 PMC7016506

[15] Aurrecoechea C, Brestelli J, Brunk BP, Dommer J, Fischer S, Gajria B, Gao X, Gingle A, Grant G, Harb OS, Heiges M, Innamorato F, Iodice J, Kissinger JC, Kraemer E, Li W, Miller JA, Nayak V, Pennington C, Pinney DF, Roos DS, Ross C, Stoeckert CJ, Treatman C, Wang H. 2009. PlasmoDB: a functional genomic database for malaria parasites. Nucleic Acids Res 37:D539–43. doi:10.1093/nar/gkn814 18957442 PMC2686598

[16] Absalon S, Robbins JA, Dvorin JD. 2016. An essential malaria protein defines the architecture of blood-stage and transmission-stage parasites. Nat Commun 7:11449. doi:10.1038/ncomms11449 27121004 PMC4853479

[17] Onn I, Aono N, Hirano M, Hirano T. 2007. Reconstitution and subunit geometry of human condensin complexes. EMBO J 26:1024–1034. doi:10.1038/sj.emboj.7601562 17268547 PMC1852836

[18] Hara K, Kinoshita K, Migita T, Murakami K, Shimizu K, Takeuchi K, Hirano T, Hashimoto H. 2019. Structural basis of HEAT-kleisin interactions in the human condensin I subcomplex. EMBO Rep 20:e47183. doi:10.15252/embr.201847183 30858338 PMC6501013

[19] Kinoshita K, Tsubota Y, Tane S, Aizawa Y, Sakata R, Takeuchi K, Shintomi K, Nishiyama T, Hirano T. 2022. A loop extrusion-independent mechanism contributes to condensin I-mediated chromosome shaping. J Cell Biol 221:e202109016. doi:10.1083/jcb.202109016 35045152 PMC8932526

[20] Schleiffer A, Kaitna S, Maurer-Stroh S, Glotzer M, Nasmyth K, Eisenhaber F. 2003. Kleisins: a superfamily of bacterial and eukaryotic SMC protein partners. Mol Cell 11:571–575. doi:10.1016/s1097-2765(03)00108-4 12667442

[21] Hassler M, Shaltiel IA, Kschonsak M, Simon B, Merkel F, Thärichen L, Bailey HJ, Macošek J, Bravo S, Metz J, Hennig J, Haering CH. 2019. Structural basis of an asymmetric condensin A TPase cycle. Mol Cell 74:1175–1188. doi:10.1016/j.molcel.2019.03.037 31226277 PMC6591010

[22] Kschonsak M, Merkel F, Bisht S, Metz J, Rybin V, Hassler M, Haering CH. 2017. Structural basis for a safety-belt mechanism that anchors condensin to chromosomes. Cell 171:588–600. doi:10.1016/j.cell.2017.09.008 28988770 PMC5651216

[23] Piazza I, Rutkowska A, Ori A, Walczak M, Metz J, Pelechano V, Beck M, Haering CH. 2014. Association of condensin with chromosomes depends on DNA binding by its HEAT-repeat subunits. Nat Struct Mol Biol 21:560–568. doi:10.1038/nsmb.2831 24837193

[24] Tane S, Shintomi K, Kinoshita K, Tsubota Y, Yoshida MM, Nishiyama T, Hirano T. 2022. Cell cycle-specific loading of condensin I is regulated by the N-terminal tail of its kleisin subunit. Elife 11:e84694. doi:10.7554/eLife.84694 36511239 PMC9797191

[25] Ouspenski II, Cabello OA, Brinkley BR. 2000. Chromosome condensation factor Brn1p is required for chromatid separation in mitosis. Mol Biol Cell 11:1305–1313. doi:10.1091/mbc.11.4.1305 10749931 PMC14848

[26] Oliveira RA, Coelho PA, Sunkel CE. 2005. The condensin I subunit barren/CAP-H is essential for the structural integrity of centromeric heterochromatin during mitosis. Mol Cell Biol 25:8971–8984. doi:10.1128/MCB.25.20.8971-8984.2005 16199875 PMC1265781

[27] Sutani T, Yuasa T, Tomonaga T, Dohmae N, Takio K, Yanagida M. 1999. Fission yeast condensin complex: essential roles of non-SMC subunits for condensation and Cdc2 phosphorylation of Cut3/SMC4. Genes Dev 13:2271–2283. doi:10.1101/gad.13.17.2271 10485849 PMC316991

[28] Knuepfer E, Napiorkowska M, van Ooij C, Holder AA. 2017. Generating conditional gene knockouts in plasmodium - a toolkit to produce stable diCre recombinase-expressing parasite lines using CRISPR/Cas9. Sci Rep 7:3881. doi:10.1038/s41598-017-03984-3 28634346 PMC5478596

[29] Voss Y, Klaus S, Guizetti J, Ganter M. 2023. Plasmodium schizogony, a chronology of the parasite’s cell cycle in the blood stage. PLoS Pathog 19:e1011157. doi:10.1371/journal.ppat.1011157 36862652 PMC9980825

[30] Nanda JS, Lorsch JR. 2014. Labeling a protein with fluorophores using NHS ester derivitization. Methods Enzymol 536:87–94. doi:10.1016/B978-0-12-420070-8.00008-8 24423269

[31] Zeeshan M, Pandey R, Ferguson DJP, Tromer EC, Markus R, Abel S, Brady D, Daniel E, Limenitakis R, Bottrill AR, Le Roch KG, Holder AA, Waller RF, Guttery DS, Tewari R. 2020. Real-time dynamics of Plasmodium NDC80 reveals unusual modes of chromosome segregation during parasite proliferation. J Cell Sci 134:jcs245753. doi:10.1242/jcs.245753 32501284 PMC7340582

[32] Brusini L, Dos Santos Pacheco N, Tromer EC, Soldati-Favre D, Brochet M. 2022. Composition and organization of kinetochores show plasticity in apicomplexan chromosome segregation. J Cell Biol 221:e202111084. doi:10.1083/jcb.202111084 36006241 PMC9418836

[33] Roux KJ, Kim DI, Raida M, Burke B. 2012. A promiscuous biotin ligase fusion protein identifies proximal and interacting proteins in mammalian cells. J Cell Biol 196:801–810. doi:10.1083/jcb.201112098 22412018 PMC3308701

[34] Meraldi P, Nigg EA. 2002. The centrosome cycle. FEBS Lett 521:9–13. doi:10.1016/s0014-5793(02)02865-x 12067716

[35] Mahajan B, Selvapandiyan A, Gerald NJ, Majam V, Zheng H, Wickramarachchi T, Tiwari J, Fujioka H, Moch JK, Kumar N, Aravind L, Nakhasi HL, Kumar S. 2008. Centrins, cell cycle regulation proteins in human malaria parasite Plasmodium falciparum. J Biol Chem 283:31871–31883. doi:10.1074/jbc.M800028200 18693242

[36] Glushakova S, Mazar J, Hohmann-Marriott MF, Hama E, Zimmerberg J. 2009. Irreversible effect of cysteine protease inhibitors on the release of malaria parasites from infected erythrocytes. Cell Microbiol 11:95–105. doi:10.1111/j.1462-5822.2008.01242.x 19016793 PMC2883916

[37] Jones ML, Kitson EL, Rayner JC. 2006. Plasmodium falciparum erythrocyte invasion: a conserved myosin associated complex. Mol Biochem Parasitol 147:74–84. doi:10.1016/j.molbiopara.2006.01.009 16513191

[38] Carter R, Miller LH. 1979. Evidence for environmental modulation of gametocytogenesis in Plasmodium falciparum in continuous culture. Bull World Health Organ 57 Suppl 1:37–52. 397008 PMC2395706

[39] Norden C, Mendoza M, Dobbelaere J, Kotwaliwale CV, Biggins S, Barral Y. 2006. The NoCut pathway links completion of cytokinesis to spindle midzone function to prevent chromosome breakage. Cell 125:85–98. doi:10.1016/j.cell.2006.01.045 16615892

[40] Steigemann P, Wurzenberger C, Schmitz MHA, Held M, Guizetti J, Maar S, Gerlich DW. 2009. Aurora B-mediated abscission checkpoint protects against tetraploidization. Cell 136:473–484. doi:10.1016/j.cell.2008.12.020 19203582

[41] Absalon S, Dvorin JD. 2021. Depletion of the mini-chromosome maintenance complex binding protein allows the progression of cytokinesis despite abnormal karyokinesis during the asexual development of Plasmodium falciparum. Cell Microbiol 23:e13284. doi:10.1111/cmi.13284 33124706 PMC8058698

[42] Robbins JA, Absalon S, Streva VA, Dvorin JD. 2017. The malaria parasite cyclin H homolog PfCyc1 is required for efficient cytokinesis in blood-stage Plasmodium falciparum. mBio 8:e00605-17. doi:10.1128/mBio.00605-17 28611247 PMC5472185

[43] Heath IB. 1980. Variant mitoses in lower eukaryotes: indicators of the evolution of mitosis. Int Rev Cytol 64:1–80. doi:10.1016/s0074-7696(08)60235-1 20815116

[44] Peterson JB, Ris H. 1976. Electron-microscopic study of the spindle and chromosome movement in the yeast Saccharomyces cerevisiae. J Cell Sci 22:219–242. doi:10.1242/jcs.22.2.219 794073

[45] Strunnikov AV, Hogan E, Koshland D. 1995. SMC2, a Saccharomyces cerevisiae gene essential for chromosome segregation and condensation, defines a subgroup within the SMC family. Genes Dev 9:587–599. doi:10.1101/gad.9.5.587 7698648

[46] Wei-Shan H, Amit VC, Clarke DJ. 2019. Cell cycle regulation of condensin Smc4. Oncotarget 10:263–276. doi:10.18632/oncotarget.26467 30719224 PMC6349450

[47] Liffner B, Absalon S. 2021. Expansion microscopy reveals Plasmodium falciparum blood-stage parasites undergo anaphase with A chromatin bridge in the absence of mini-chromosome maintenance complex binding protein. Microorganisms 9:11. doi:10.3390/microorganisms9112306 PMC8620465 34835432

[48] Piazza I, Haering CH, Rutkowska A. 2013. Condensin: crafting the chromosome landscape. Chromosoma 122:175–190. doi:10.1007/s00412-013-0405-1 23546018

[49] Kimura K, Cuvier O, Hirano T. 2001. Chromosome condensation by a human condensin complex in xenopus egg extracts. J Biol Chem 276:5417–5420. doi:10.1074/jbc.C000873200 11136719

[50] Giet R, Glover DM. 2001. Drosophila aurora B kinase is required for histone H3 phosphorylation and condensin recruitment during chromosome condensation and to organize the central spindle during cytokinesis. J Cell Biol 152:669–682. doi:10.1083/jcb.152.4.669 11266459 PMC2195771

[51] Pease BN, Huttlin EL, Jedrychowski MP, Talevich E, Harmon J, Dillman T, Kannan N, Doerig C, Chakrabarti R, Gygi SP, Chakrabarti D. 2013. Global analysis of protein expression and phosphorylation of three stages of Plasmodium falciparum intraerythrocytic development. J Proteome Res 12:4028–4045. doi:10.1021/pr400394g 23914800 PMC5292867

[52] Liffner B, Cepeda Diaz AK, Blauwkamp J, Anaguano D, Frolich S, Muralidharan V, Wilson DW, Dvorin JD, Absalon S. 2023. Atlas of Plasmodium falciparum intraerythrocytic development using expansion microscopy. Elife 12:RP88088. doi:10.7554/eLife.88088 38108809 PMC10727503

[53] Simonetti AB. 1996. The biology of malarial parasite in the mosquito--a review. Mem Inst Oswaldo Cruz 91:519–541. doi:10.1590/s0074-02761996000500001 9137738

[54] Jones ML, Cottingham C, Rayner JC. 2009. Effects of calcium signaling on Plasmodium falciparum erythrocyte invasion and post-translational modification of gliding-associated protein 45 (PfGAP45). Mol Biochem Parasitol 168:55–62. doi:10.1016/j.molbiopara.2009.06.007 19576251 PMC2754074

[55] Needleman SB, Wunsch CD. 1970. A general method applicable to the search for similarities in the amino acid sequence of two proteins. J Mol Biol 48:443–453. doi:10.1016/0022-2836(70)90057-4 5420325

[56] Robert X, Gouet P. 2014. Deciphering key features in protein structures with the new Endscript server. Nucleic Acids Res 42:W320–4. doi:10.1093/nar/gku316 24753421 PMC4086106

[57] Lambros C, Vanderberg JP. 1979. Synchronization of Plasmodium falciparum erythrocytic stages in culture. J Parasitol 65:418–420. 383936

[58] Saul A, Myler P, Elliott T, Kidson C. 1982. Purification of mature schizonts of Plasmodium falciparum on colloidal silica gradients. Bull World Health Organ 60:755–759. 6758971 PMC2536039

[59] Collins CR, Hackett F, Strath M, Penzo M, Withers-Martinez C, Baker DA, Blackman MJ. 2013. Malaria parasite cGMP-dependent protein kinase regulates blood stage merozoite secretory organelle discharge and egress. PLoS Pathog 9:e1003344. doi:10.1371/journal.ppat.1003344 23675297 PMC3649973

